# Effects of synthetic cannabinoids on psychomotor, sensory and cognitive functions relevant for safe driving

**DOI:** 10.3389/fpsyt.2022.998828

**Published:** 2022-09-26

**Authors:** Vasco Orazietti, Giuseppe Basile, Raffaele Giorgetti, Arianna Giorgetti

**Affiliations:** ^1^Department of Excellence of Biomedical Sciences and Public Health, Marche Polytechnic University of Ancona, Ancona, Italy; ^2^Istituto di Ricovero e Cura a Carattere Scientifico (IRCCS) Galeazzi Orthopedics Institute, Milan, Italy; ^3^Unit of Legal Medicine, Department of Medical and Surgical Sciences, University of Bologna, Bologna, Italy

**Keywords:** psychomotor performance, driving ability, driving impairment, synthetic cannabinoids, Spice

## Abstract

Recreational use of Synthetic Cannabinoids (SCs), one of the largest groups of New Psychoactive Substances (NPS), has increased globally over the past few years. Driving is a structured process requiring the cooperation of several cognitive and psychomotor functions, organized in different levels of complexity. Each of these functions can be affected when Driving Under the Influence (DUI) of SCs. In order to reduce the likelihood of SC-related road accidents, it is essential to understand which areas of psychomotor performance are most affected by these substances, as well as the severity of impairment. For this purpose, a multiple database- literature review of recent experimental studies in humans and animals regarding the psychomotor effects of SCs has been performed. Despite the many limitations connected to experimental studies on humans, results showed a consistency between animal and human data. SCs appear to impair psychomotor performance in humans, affecting different domains related to safe driving even at low doses. Cases of DUI of SC have been repeatedly reported, although the exact prevalence is likely to be underestimated due to current analytical and interpretative issues. For this reason, an accurate physical examination performed by trained and experienced personnel has a primary role in recognizing signs of impairment in case of strong suspicion of SC consumption. The identification of a suspected case should be followed by reliable laboratory examination.

## Introduction

Synthetic cannabinoids (SCs) are a large group of new psychoactive substances (NPS), chemically designed to mimic the effects of the natural cannabis (1), although generally with higher affinity to the cannabinoid receptors (CB_1_ and CB_2_) and potency compared to delta-9-tetrahydrocannabinol (THC). Recreational use of SCs has increased globally over the past few years, due to availability, easy trafficking, perceived legal status and lack of detection at routine screening (2–4). The spreading of SCs has caused growing concern (5), requiring the attention of the European Monitoring Center for Drugs and Drug Addiction, which has so far monitored more than 224 compounds (6). The continuous introduction into the market of NPS of new and powerful synthetic molecules makes it difficult for forensic toxicologists to “keep up with the times” (7, 8). Indeed, these substances are rarely detected by common screening methods and require target methodologies (9), which have to be constantly updated. Furthermore, as for other NPS, not all the available molecules are currently regulated under national or international legislations, e.g., schedule 1 substances in the United States, the law on NPS (Neue-psychoaktive-Stoffe-Gesetz or NpSG) in Germany, the DPR 309/90 and updates in Italy, making some SCs legal. Typical symptoms following the consumption of SCs include nausea, vomiting, tachycardia, agitation, psychomotor agitation, seizures (10), but also respiratory depression, which have been reported in cases of fatal and non-fatal intoxications (11, 12). Feeling of euphoria, being “high” and “stimulated,” but also somnolence and minimal dizziness or confusion were reported by subjects consuming SCs (13, 14). Several cases of driving under the influence (DUI) involving NPS have been also reported in literature (15–17). Between January 2019 and April 2020, 62% out of 670 NPS toxicology case reported to the UNODC were classified as DUI (18). Driving is a structured process that requires the cooperation of multiple cognitive and psychomotor functions, organized in different levels of complexity (19). Each of these cognitive and psychomotor functions can be affected by ingestion, inhalation, absorption, or injection of drugs or medications (20) including SCs. While several studies are focused on alcohol and classic drugs effects on driving ability, there is a lack of knowledge regarding the effects related to SCs. In order to reduce the likelihood of SC-related road accidents, it is fundamental to understand which areas of psychomotor performance are most affected by these substances as well as the severity of impairment. To this scope, in the present study, a literature review regarding the psychomotor effects connected to SCs has been performed.

## Materials and methods

A first literature search was conducted using the most common databases (PubMed, Scopus, Web of Science), focusing on both cognitive and executive functions needed for safe driving. Then, a search focused on the SC-related driving skills impairment was performed by combining phrase keywords including “synthetic cannabinoids” OR “SC” OR “Spice” AND one of the followings: “psychomotor performance,” “driving skills,” “driving ability,” “executive functions,” “motor function,” “memory,” “attention.” The following studies were included: preclinical and clinical trials, randomized controlled trials, studies performed on humans or animals published in the last 10 years (from 2012 to 2022). Real cases of DUI were also included in the literature revision, but only when SCs were confirmed on blood and data was extractable. This temporal limit was set in order to provide a review of the most recent evidence on the topic. Non-systematic reviews, studies where SCs were administered together with other substances, intoxication cases with no mention of DUI and papers with no full-text available were excluded from this study. The search was not restricted to English language documents. Results were then summarized in two separate tables for a better data overview. For studies performed on animals, functions examined, authors, years of publication, testing methods (i.e., type of tests administered), treatment parameters (i.e., substance and doses) and main findings were extracted from the included papers. For studies performed on humans, similarly the following data was extracted: functions examined, authors, years of publication, number of participants, testing methods, treatment parameters, main findings. For DUI cases, author, number of cases, age and sex of the participant, results of the toxicological analyses on blood for SCs and for other substances as well as the impairment in psychomotor performances relevant for driving, as emerged from clinical examination/police reports, drug experts' evaluation, etc., were extracted.

## Results

After the search with the selected terms, more than 500 hits, duplicates excluded, were identified. Thirty-five studies met the inclusion criteria, eight of which were performed on humans (21–28) and 27 on animals (mice) (29–55). Detailed information is described in Table 1 and in Table A of Supplementary material for human and animal studies collected, respectively. Additionally, 13 articles reported cases of DUI of SCs (56–68). Papers focused on humans tested several domains related to safe driving, which included cognitive and integrative functions (visuospatial and executive functions, attention, memory, planning, information processing speed, response inhibition), emotional processing and motor performance. All these domains were examined using validated neuropsychological tests. Three of the eight papers collected were randomized controlled trials in which vapor inhalation of JWH-018 or placebo were administered, five studies used test administration only, in the absence of a control. Administered doses of JWH-018 were fixed (2 or 3 mg) or calculated according to body weight (75 μg/kg). The number of subjects examined in each study ranged from 6 to 145 with a median of 48. Three papers involved occasional users, three chronic SCs users compared with regular cannabis users and non-users, and two included chronic SCs users and non-users. Results showed impairment in motor coordination, attention, working and long-term memory and lower speed-accuracy efficiency and response speed, together with impaired executive, cognitive and visuospatial functions, response inhibition and information processing in SCs users (more details in Table 2). Experiments performed on animals involved mice in all the studies collected. The included articles focused primarily on memory (working, reference, spatial and recognition memory) sensorimotor performance and locomotor activity. The latter was the most commonly studied function, evaluated in 24 out of 27 studies, even when no other psychomotor performances were considered. Two studies were performed on adolescent animals, in order to assess the effects of SCs on learning and behavior once they become adults. SCs were administered by intraperitoneal injections or by exposition to smoke from a mixture containing the compounds. Impairment in psychomotor performance was described in all the studies, especially regarding locomotor activity and sensorimotor performance. DUI cases reported 1–24 cases (mean 8), for a total of 53. Subject had a mean age of 25 years (from 16 to 48) and were predominantly male, except for 2 cases. Other substances, beside SCs, were detected in 35 cases. When quantifies, SCs were in the low nanogram range, 0–3 ng/ml, though some exceptions were reported (maximum level was described for JWH-122: 73 ng/ml). The evaluation of subjects also included a wide range of parameters, including movements, coordination, balance, speech, eye movements (pupils and convergence), mood, reaction times, and type of accident/driving offense as well as the performance at tests such as Romberg, finger-to-nose (FTN) and finger-to-finger (FTF), walk-and-turn (WAT) and one leg stand (OLS) tests. Detailed information is described in Table 2.

**Table 1:** Studies performed on humans.

**Functions**	**Ref**.	**P. number**	**Testing methods**	**Treatment**	**Results**	**Type of users**
Concentration; memory; language; visuoconstructional skills; conceptual thinking; calculation; orientation; executive functions; attention speed; motor speed; visual search speed; ordering skills; mental flexibility; persistence; response inhibition; susceptibility to interference; verbal attention; continuous and selective attention	(22)	145	Montreal Cognitive Assessment (MOCA) Test, Verbal Memory Processes Test (VMPT), Clock Drawing Test, Cube Drawing Test, Trial Making Test, Verbal Fluency Test, Digit Span Test, Continuous Performance Test (CPT), Stroop Test, Go/No-Go Test	–	More severe impairments in attention, memory, executive and visuospatial functions in the SC group than in the cannabis and the healthy control groups	Chronic SCs users, chronic cannabis users, non-users
Executive functions; emotional processing; depression and anxiety traits	(28)	122	Beck depression inventory (BDI), Spielberg state-trait anxiety inventory (STAI), Stroop word-color task, N-back task, free-recall memory task	–	In the SC group impairments in working/long-term memory, response inhibition. Higher ratings of depression and anxiety	Chronic SCs users, chronic cannabis users, non-users
Working memory; response inhibition; depression and anxiety traits	(27)	33	Working memory N-back task, response-inhibition Go-No-Go task, Beck depression inventory (BDI), State-trait anxiety inventory (STAI)	–	In SC group higher ratings on the BDI, STAI compared with control participants. SC impaired performance on the N-back task, but not on the Go-No-Go task	Chronic SCs users, non-users
Cognitive performance; attention; executive functions; planning; memory; subjective experience	(25)	6	Digit symbol substitution task (DSST), Critical tracking test (CTT), Divided attention task (DAT), Stop signal task (SST), Tower of London (TOL), Spatial memory task (SMT), Subjective high, Profile of moods states (POMS), Bowdle visual analog scales, marijuana craving questionnaire (MCQ), Sensitivity to Cannabis Reinforcement Questionnaire (SCRQ), Clinician-administered dissociative States Scale (CADSS)	Inhalation JWH-018 (2–3 mg) or placebo	JWH-018 impaired motor performance (CTT), divided attention (DAT) and response inhibition (SST), particularly after the 2 mg dose. Executive functioning (TOL), spatial memory (SMT), speed and information processing (DSST) were not affected by JWH-018	Occasional cannabis users
Cognitive performance; attention; executive functions; memory; subjective experience	(26)	17	Digit symbol substitution DSST, critical tracking task CTT, divided attention task DAT, stop signal task SST, Tower of London TOL, spatial memory task [SMT], Profile of Mood States [POMS], Bowdle visual analog scales, Marijuana Craving Questionnaire [MCQ], Sensitivity to Cannabis Reinforcement Questionnaire [SCRQ], Clinician-Administered Dissociative States Scale [CADSS]	Inhalation of JWH-018 (2–6.2 mg, average 3.95 mg) or placebo	Lower CTT, SST, SMT scores in CS group. No significant effects in DAT, TOL, and DSST. Large variability in the subjective response to the drug	Occasional cannabis users
Visuospatial functions; executive functions; attention; working memory; speaking; abstract thinking; hand preference; motor speed; information processing speed	(23)	63	Montreal Cognitive Assessment (MOCA) test, Edinburgh Handedness Inventory (EHI), Finger-Tapping Test (FTT), Adult Memory and Information Processing Battery-B form (AMIPB-B)	–	SC group scored worse in AMIPB-B, MOCA, and FTT	Chronic SCs users, non-users
Executive functions; emotional processing; depression and anxiety traits	(24)	94	N-back task, Go/No-Go task, Wisconsin Sorting Card-like Task (WSCT), emotional face recognition task, questionnaires of depression, anxiety and schizotypal traits	–	SC group scored worse on the N-back working-memory task and WSCT cognitive flexibility task; showed greater schizotypal traits and symptoms and higher scores on depression and state-trait anxiety measures	Chronic SCs users, chronic cannabis users, non-users
Motor coordination; attention; memory; speed-accuracy efficiency; response speed; motor impulsivity; reflection impulsivity; planning	(21)	24	Critical Tracking Test (CTT), VAS, Divided Attention Task (DAT), Spatial Memory Task (SMT), Stop Signal Task (SST), Matching Familiar Figures Test (MFFT), Digit Symbol Substitution Task (DSST), Tower of London (TOL), subjective high	Inhalation of JWH-018 (75 μg/kg plus booster dose of 50 μg/kg, average 5.52 mg) or placebo	Maximum subjective high 30 min after administration, maximum blood concentration after 5 min (8 ng/mL). Impaired motor coordination, attention, memory. Lower speed-accuracy efficiency, slowed response speed in a 4-h window after administration, most strongly within the first 2.5 h	Occasional cannabis users

Ref., reference number; P. number, participants' number.

**Table 2:** Cases of DUI of SCs.

**Ref**.	**Cases**	**Age, sex**	**SC blood levels (ng/ml)**	**Other drugs**	**Psychomotor performances relevant for driving**
Lemos (56)	1	22, M	XLR-11: 1.34	–	Slow body movements and coordination, slow, low and mumbled speech, lethargy, droopy eyelids, stiff and rigid muscle tone, lack of convergence, impaired OLS, unable to maintain balance at the WAT, no coordination at the FTN test
Yeakel and Logan (57)	12	–	JWH-018: 1.1	–	WAT: arm raising, swayed, improper turn. OLS: flexed foot, arm raising. Slow reaction times
		18	JWH-018: 0.24	–	WAT: arm raising, improper turn. OLS: arm raising. Romberg: eye flutters
		22	JWH-018: 9.9 JWH-250: 2.7	–	HGN
		25	JWH-018: pos	–	OLS: swaying movements, arm raising, tremors. Romberg: rapid eyelid and hand tremors
		18	JWH-018: pos	–	WAT: loss of balance. OLS: incorrect counting, swaying movements, leg tremors. Romberg: leg, eye tremors
		31	AM-2201: 1.4 JWH-081: 0.12 JWH-122: 2.5 JWH-210: 0.10	Caffeine, theobromine, nicotine and cotinine	WAT and OLS: swaying movements, arm raising. Romberg: eye tremors
		27	JWH-018: 0.1 AM-2201: 0.43 JWH-122: pos JWH-210: pos	–	Romberg: eye tremors
		21	AM-2201: 3.1 JWH-250: 0.38	–	WAT: leg, body tremors. OLS and Romberg: swaying movements, tremors
		26	AM-2201: 0.94	–	–
		18	AM-2201: 3.6	–	WAT: arm raising, leg tremors. OLS: swaying movements, arm raising, foot down Romberg: swaying movements, leg tremors
		21	AM-2201: 2.8 JWH-081: pos JWH-122: pos JWH-210: pos	–	HGN. WAT: imbalance with near-fall
		19	AM-2201: 4.0 JWH-210: pos	–	WAT and OLS: tremors
Musshoff et al. (61)	7	18, M	AM-2201: 4.6 JWH-018: 0.17	–	Inability to follow instructions, slow movements, confusion, disorientation, slurred speech, nearly unconsciousness
		14, F	JWH-210: 4.0 JWH-122: 0.33	–	Cycling in wavy lines. Instability, slurred speech, dizzy mind
		20, M	JWH-018: 1.7 JWH-122: 7.6 JWH-210: 4.4 AM-2201: 0.31	–	Vestibular disorder, disturbance of fine motor skills, doubtful FTF, delayed pupils' reaction to light
		29, M	JWH-210: 6.2 JWH-122: 1.0	–	Delayed pupils' reaction to light, dizzy mind, retarded behavior
		21, M	JWH-018: 0.52 JWH-122: 0.26 JWH-210: 0.66	–	Delayed reactions, retarded movements, dizzy mind, no pupils' reaction to light, nervousness and laziness
		21, M	JWH-307: 1.1	Ethanol	Fast driving, run off the road.
		22, M	JWH-018: 1.0 JWH-122: 28 JWH-210: 2.5 AM-2201, JWH-307, MAM-2201, UR-144 pos	–	Retarded movements, nervousness, delayed pupils' reaction to light
Louis et al. (62)	18	22, M	UR-144: pos	–	Speed, lane travel. Poor coordination, tremors, altered WAT, OLS
		22, M	UR-144: pos	–	Lane travel. Poor coordination, slurred speech, lack of convergence, tremors, altered WAT, OLS
		25, M	UR-144: pos	–	Unconscious, accident. Shaking coordination, slurred speech, tremors
		42, M	UR-144: pos	–	Lane travel. Poor coordination, slurred speech, lack of convergence, altered WAT, OLS
		29, M	UR-144: pos	–	Erratic driving. Poor coordination, droopy eyelids, slurred speech, tremors, altered WAT, OLS
		30, M	UR-144, XLR-11: pos	–	Speed, lane travel. Poor coordination, slurred speech, tremors, altered WAT, FTN
		22, M	UR-144, XLR-11: pos	–	Lane travel. Lack of convergence, tremors, altered OLS, FTN
		23, M	UR-144, XLR-11, AM-2201, JWH-018, JWH-022: pos	–	Lane travel. Poor coordination, slurred speech, altered WAT, OLS
		30, M	UR-144, XLR-11: pos	–	Accident. Lack of convergence, tremors, altered OLS, FTN
		25, M	UR-144: pos	–	Lane travel. Swaying movements, slurred speech, lack of convergence, tremors, altered WAT, OLS
		27, M	XLR-11: pos	–	Speed, lane travel, accident. Slurred speech
		27, M	XLR-11: pos	–	Speed, lane travel, driving on curb, accident. Poor coordination, slurred speech, lack of convergence, tremors, altered WAT, OLS, FTN
		30, M	XLR-11: pos	–	Moving violation. Altered WAT, OLS
		17, M	XLR-11: pos	–	Lane travel. Poor coordination, altered WAT
		22, M	XLR-11: pos	–	Unconscious, accident. Poor coordination, slurred speech, altered WAT, OLS
		19, M	XLR-11: pos	–	Poor coordination, slurred speech, lack of convergence, tremors, altered WAT, OLS, FTN
		22, M	XLR-11: pos	–	Driving on curb. Slow coordination, droopy eyelids, tremors, altered WAT, OLS, FTN
		23, M	XLR-11: pos	–	Driving in the wrong way. Poor coordination, lack of convergence, tremors, altered WAT, OLS, FTN
Tuv et al. (63)	16	48, M	JWH-081: 0.19	Amphetamine, methamphetamine, BDZ	Mild impairment
		35, M	JWH-250: 0.47	Amphetamine, methamphetamine, BDZ	Moderate impairment
		29, M	JWH-018: 0.24	Amphetamine, methamphetamine, THC	No impairment
		17, M	AM-2201: 0.07	Methamphetamine, BDZ	Mild impairment
		20, M	RCS-4: 1.0	BDZ, THC	Mild impairment
		25, M	JWH-122: 1.2	THC	No impairment
		31, M	AM-2201: 0.25	methamphetamine, BDZ, THC	–
		35, F	JWH-018: 0.13	Amphetamine, methamphetamine, BDZ, THC	Mild impairment
		30, M	JWH-018: 0.10	BDZ, THC	–
		41, M	AM-2201: 0.28	BDZ, THC, methadone	–
		33, M	JWH-018: 0.46	Methamphetamine, BDZ	No impairment
		29, M	JWH-122: 0.50	Ethanol, THC, LSD	Traffic accident
		27, M	JWH-122: 1.67	THC, ketamine, LSD	Traffic accident
		26, M	JWH-018: 0.08	BDZ, THC	Traffic accident. Severe impairment
		30, M	AM-2201: 0.4	BDZ, methylphenidate, THC	Severe impairment
		16, M	AM-2201: 1.33	BDZ, THC	Mild impairment
Kaneko (64)	6 (others with no blood confirmation)	25, M	AM-232: pos	–	Speed driving and accident. Blurred vision, abnormal posture, no crash memory
		24, M	5F-PB-22: 0.27	–	Lane travel and frontal collision. Loss of consciousness, no crash memory
		38, M	5F-PB-22: 0.39	–	Driving on curb, continued accelerating after impact with pedestrians, moving the steering wheel and gear lever in a stereotyped manner, no crash memory
		37, M	FUB-PB-22: 1.74	–	Car accident. Slow movements, impaired consciousness, excited and agitated, no crash memory
		28, M	5F-AMB: pos	–	Ignoring red light and collision. Motionless, unresponsiveness, no crash memory
		26, M	5F-AMB: 0.07 FUB-PB-22: 0.79	–	Car accident. Impaired consciousness, no crash memory
Kleis et al. (65)	1	29, M	5F-MDMB-PICA metabolite: pos	–	Traffic offense. Balance deficits, staggering, pupils' unresponsiveness to light, lethargy slow reactions, aggressive behavior, numbness
Jaenicke et al. (66)	12	24, M	JWH-122: 73.05	–	–
		19, M	JWH-250: 0.91 JWH-122: 9.53	–	Delayed pupils' reaction to light, eyelid twitching, impairments of FTF and Romberg tests, change in time perception
		46, M	JWH-015: pos	Ethanol	–
		18, M	JWH-250: 0.21	THC	Agitation, eyelid twitching, impairment of FTF, knees slightly tremulous, unsteady mood
		25, M	JWH-250: 2.94 JWH-015: pos JWH-018: 0.75 JWH-122: 1.3	THC	Slight delayed pupils' reaction to light, impairment of FTF
		21, M	JWH-122: 0.11	THC	Delayed pupils' reaction to light, light eyelid twitching, tremor, impairment of Romberg test
		24, M	JWH-122: pos	THC	Delayed pupils' reaction to light, agitation, vertical nystagmus, eyelid twitching, unsteadiness, impairments of FTF and Romberg tests, paranoia and delusions
		20, M	JWH-122: 0.44 JWH-210: 1.06	Amphetamine	Delayed pupils' reaction to light, eyelid twitching, HGN, impairments of FTF and Romberg tests, OLS unsteady
		19, M	JWH-122: 0.35	Ethanol, amphetamine	Inconspicuous mood
		20, M	JWH-122: 1.91	THC, morphine	Agitation, no reaction to light, eyelid twitching, trismus
		36, M	JWH-016: pos	Ethanol, cocaine, methadone, BDZ	Nystagmus, unsteadiness, impairment of FTF and FTN, retarded/delayed pupils' reaction, dizzy mind, slurred speech
		38, M	JWH-250: 2.88	Ethanol, tramadol, BDZ	Nystagmus, FTF and FTN impairment, depressive mood
McCain et al. (67)	1	45, M	5F-ADB metabolite: 26.37	–	Driving against the traffic and swerving the vehicle into a roadside guard rail. Loss of consciousness, nystagmus, WAT and OLS impaired
Kraemer et al. (68)	1	26, M	5F-ADB: 0.19	–	Erratic driving, lane travel. Mood alterations, retarded/delayed pupils' reaction
Peterson and Couper (58)	24	22, M	AB-PINACA: 27.8	–	Traffic accident. Confusion, slurred speech, shaking, impaired WAT and OLS
		Same subject	AB-PINACA: 10	–	Vehicle stopped in the roadway. Inability to stand.
		Same subject	AB-CHMINACA: 9.1	–	Erratic driving
		Same subject	AB-CHMINACA: 4.5	–	Slow lethargic movements, inability to maintain balance
		–	AB-PINACA: 2.6 AM-2201: 2.1 JWH-210: 0.2	–	Lack of convergence, slightly impaired WAT and OLS
		–	AB-PINACA: 4.6	–	Lack of convergence, impaired WAT and OLS
		–	AB-PINACA: 5.7	–	VGN, lack of convergence, impaired WAT and OLS
		–	AB-PINACA: 8.3	–	OLS slight impairment
		–	AB-PINACA: 9.1	–	No impairment
		–	AB-PINACA: 9.1	–	Lack of convergence, impaired WAT and OLS
		–	AB-PINACA: > 10	–	Lack of convergence, eyelid tremors, impaired WAT and OLS
		–	AB-PINACA: > 10	–	–
		–	AB-PINACA: > 10	–	Lack of convergence, eyelid tremors, impaired WAT and OLS
		–	AB-PINACA: 41.3	–	Lack of convergence, impaired WAT and OLS
		38, M	AB-CHMINACA: 2.3	–	Vehicle blocking the roadway. Lethargy, confusion, imbalance, HGN, lack of convergence, impaired WAT and OLS
		19, M	AB-CHMINACA: 7.1	Diphenhydramine	Asleep at wheel. Slurred speech, swaying movements, instability
		Same subject	AB-CHMINACA: 7.1	Diphenhydramine	Poor driving. Impaired WAT and OLS
		–	AB-CHMINACA: 1	THC	HGN
		–	AB-CHMINACA: 2.4	–	HGN, VGN, lack of convergence
		–	AB-CHMINACA: 3.5	–	Impaired WAT and OLS
		–	AB-CHMINACA: 4.4 UR-144: 0.3 5F-ABPINACA: 0.4	–	HGN, WAT, and OLS slightly impaired
			AB-CHMINACA: 6.3	–	No impairment
			AB-CHMINACA: 7.3	–	Eyelid tremors, impaired WAT and OLS
			AB-CHMINACA: 9.5		VGN, impaired WAT
Fels et al. (59)	4 (others with traces only)	23, M	5F-ADB: 1.4	BEC, methamphetamines	Delayed pupils' reaction to light, shaking
		22, M	5F-Cumyl-PICA: pos	THC	Delayed pupils' reaction to light, imbalance, nervousness, restlessness
		19, M	MDMB-CHMICA: 0.04	THC	Eyelid twitching, lack of concentration, shaking
		28, M	5F-MDMB-PICA: 0.04 Cumyl-PEGACLONE: 0.75	THC, BEC	Slurred speech, euphoria, restlessness
Adamowicz and Lechowicz (60)	1	19, M	UR-144: 14.6	–	Car accident. Hyperactive behavior, sluggish pupils' reaction to light, gait abnormalities, staggering

DUI, driving under the influence; SCs, synthetic cannabinoids; Ref., reference number; M, male; F, female; pos, positive; BDZ, benzodiazepines; BEC, benzoylecgonine; THC, delta-9-tetrahydrocannabinol; OLS, one leg stand test; WAT, walk-and-turn test; FTF, finger-to-finger test; FTN, finger-to-nose test; HGN, horizontal nystagmus; VGN, vertical nystagmus. Impairment was defined in the presence of more than 1 clue.

## Discussion

Our study aimed at reviewing the current literature regarding the effects of SCs on cognitive and psychomotor functions involved in driving ability. As emerged from the present literature, the number of studies investigating and demonstrating such effects is still limited, so that the understanding of the effects of SCs on psychomotor performance remains mainly based on animal data or, in humans, on self-reports and overdoses (7). Although animal data were more abundant and assessed a wide range of performances relevant for driving, it has to be reminded that the inference from animal models to humans presents several limitations and should be performed with caution (69). Indeed, dose translations, as demonstrated in clinical trials, cannot be based only on the weight, but would require a careful evaluation of more data (including oxygen use, basal metabolism, caloric expenditure, distribution and blood volume, plasma proteins, and renal function) (70). To partially overcome this limit, human equivalent doses should be calculated on the basis of the body surface area, although further research is needed to provide more appropriate conversions. Beside doses, the evaluation of psychomotor function in mice are different and might not correspond to the experimental tests performed on humans, which again might not reproduce the impairment in real-driving. As demonstrated for other compounds and NPS (69), the limitedness of studies on humans is partially connected to the ethical challenges related to the administration to healthy volunteers of potent compounds with possible unpredictable effects on one hand. Indeed, in order to reduce potential negative effects, the randomized controlled trials included in the present study typically involved “occasional users” and not naïve subjects (21, 25, 26). This might lead to an underestimation of the effects of SCs due to a developed tolerance. Moreover, aiming at predicting effects and reducing harm to subjects, in this kind of studies JWH-018, one of the first generation of SCs, was administered. However, JWH-018 has been substantially abandoned as recreational drug since many years, and replaced by novel generation molecules, often characterized by higher potency (71, 72), so that the effects on driving of the more widespread and potent SCs remains unknown. Only a few randomized control trials (RCTs) performed on humans were found and these studies involved a small sample size, so that it is hard to make generalizations to the whole populations. Many factors remain largely unexplored, e.g., the impact of co-administered drugs. Finally, as underlined by Theunissen et al. (21, 26), blood levels in controlled studies (~8 ng/ml of JWH-018) are far below the concentrations reported in real cases, when other factors, such as multiple administrations, different administration routes, and tolerance, also have an impact on the severity of the impairment as well as on the duration of the effects. The large number of molecules available, the absence of routine toxicological screenings effective for their detection, the lack of labeled analytical reference standards and the high potency of these compounds, which often leads to very low concentration in tissues, are factors that make the identification and quantification of SCs, in real driving cases and epidemiological studies, complex and strictly dependent on laboratory techniques and instrumentation (8). These factors could inevitably lead to an underestimation of DUI related to SCs consumption due to false negative results. Currently, liquid chromatography-tandem mass spectrometry (LC-MS/MS) together with Liquid chromatography with quadrupole time of flight mass spectrometry (LC-QTOF-MS) represent the methods of choice for the recognition of NPS and SCs in multiple biological matrices, offering sensitive and specific identification and allowing for their quantification (73–79). LC-QTOF-MS might lack the sensitivity required to detected very low SCs concentrations, as expected given the limited half-life of compounds and the potential delay in blood collection. However, it allows an untargeted screening involving emerging compounds (80). By using LC-QTOF-MS coupled with LC-MS/MS, Fels et al. found the presence of SCs in 12 of 837 blood samples collected from suspected German DUI cases in 2017–2018 (59). Similar results are described in literature from samples obtained during police checks or following road accidents (56, 57, 60, 61, 63), making it possible to ascertain that SCs consumption while driving is a widespread reality. For this reason, it is important to request in-depth examinations whenever there is a suspicion of SCs intake, even when first routine examinations test negative. Another interesting topic of debate consists in the correlation between toxicological findings and impairment in driving abilities. Blood has always been the matrix of choice for DUI cases, because it usually correlates with the effects at the central nervous system and with the driving impairment. However, analytical inconsistencies and different timings of sampling (being blood not collected on site), might impair the opportunity to compare cases of DUI due to SCs. The blood or alternative matrix correlation to driving abilities for SCs has yet to be better evaluated (81).

### SCs and psychomotor functions in animals

One of the advantages of animal studies is certainly the possibility of testing a wider panel of compounds. Indeed, in the studied included in the present review, synthetic cannabinoids pertaining to the so-called “third generation” of SCs were tested, i.e., composed by a four-substructure pattern (tail–core–link–ring) resembling JWH-018, but with a substituent at any of these substructures (82).

The influence of SCs on spontaneous locomotor activity was proven by static (bar test) and dynamic conditions (drag and accelerod test) with the strongest reduction in the distance traveled with JWH-018 at 6 mg/kg (45–47). At the accelerod tests, 5F-AKB-48 induced a prolonged and significant locomotion impairment at doses of 3 mg/kg, while the non-fluorinated analog required higher doses (6 mg/kg) to produce only transient effects (30). Hypomobility was also shown by nose-only exposure to a mixture of JWH-018 and other SCs (50). High doses of AB-FUBINACA (3–4 mg/kg) and PB-22 (0.4 mg/kg), produced a dose-dependent decrease of locomotor activity in the staircase paradigm, while AB-CHMINACA showed effects at 0.5–1 mg/kg (49). Interestingly, systemic injection, but not intracerebroventricular injection of 5F-AMB produced impairment of locomotor activity, suggesting a peripheral effect (41). Sensorimotor studies mainly focused on visual, acoustic and tactile responses (30, 46, 47). The halogenated compounds tested by Bilel et al. (JWH-018-CL, JWH-018-Br, and AM-2201) altered sensory and motor parameters in a dose-dependent manner, though JWH-018-Br appeared less potent than the others in tactile responses (46). AKB-48 and 5F-AKB-48, 5F-ADBINACA, AB-FUBINACA, and STS-135 affected the startle response to visual, acoustic, and tactile stimuli in mice but were less effective than JWH-018 (30, 47). In the studies of Canazza et al. (30) and (47) the administration of JWH-018, AKF-48, 5F-AKB-58, 5F-ADBINACA, AB-FUBINACA, and STS-135 induced sensorimotor alterations as well as convulsions, hyperreflexia, tail elevation and aggressive behavior. The memory function and spatial learning ability were evaluated by Schreiber et al. (49) though the Y-maze paradigm, showing an impairment under high doses of AB-FUBINACA (4 mg/kg) and even low doses of AB-CHMINACA (0.125 mg/kg) and PB-22 (0.05 mg/kg). Recognition memory was impaired by the administration of 5F-AMB but only in the acquisition, and not in the recall (41), suggesting that subjects might be able to recall previously experienced environmental contents. Cha et al. showed no effect of JWH-081 and JWH-210 (both at 0.1–5 mg/kg) on learning and memory (51). On the contrary, Barbieri et al. demonstrated an effect of JWH-018, JWH-018-Br and JWH-018-Cl on working memory as observed by the novel object recognition test (NOR) (48). Spatial memory, tested though the Morris water maze test, was impaired after 0.25 mg/kg intraperitoneal injection of CP55.940 (32). Similar results have been reported from Basavarajappa et al., with 1.25 mg/kg of JWH-081 impairing memory in the NOR test and in the Y maze (31). Recognition memory impairment in the NOR was also reported together with “tetrad effects” by Canizzaro et al. after 3 mg/kg APICA intraperitoneal injection (54). Memory impairment at the Morris water maze was also described in adult mice treated with WIN55212.2 during adolescence (37). Musa et al. reported a higher anxiety-like and compulsive-like state in adulthood after a 5F-MDMB-PICA exposure during adolescence (39).

Taken together, these results allowed to confirm an effect of SCs on several functions (locomotor activity, spatial memory, sensorimotor functions) on animals. Moreover, these studies suggest that, although all SCs have an effect on cannabinoid receptors and pertain to the same NPS group, the nature and severity of their effects might differ from one molecule to the other.

### SCs and psychomotor functions in humans

Despite the limitations connected to epidemiological and experimental studies, it has to be noted that all the collected articles described some grade of impairment in either cognitive or psychomotor performances after the administration of SCs. Particularly, JWH-018 at doses from 2 mg impaired motor coordination as evaluated by the Critical Tracking Test and attention as demonstrated by Divided Attention Task and Stop Signal Task (21, 25, 26). By increasing the dose to 75 μg/kg (average dose 3.95 mg) and by applying a booster of 50 μg/kg (average dose 5.52 mg) when no subjective response was seen, spatial memory and impulsivity (as demonstrated by the Matching Familiar Figures Test) were also affected (21, 26). The latter is an important function, corresponding to the ability of problem-solving functions, which could be relevant for operating a vehicle and particularly for the avoidance of crashes. Other executive decision-making or attentional tests, previously shown to be sensitive for cannabis, such as the Digit Symbol Substitution Task or the Tower of London, were not affected by JWH-018 administration, with the maximal doses reported (21, 25, 26). Impairment was mostly comparable to the administration of 250–300 μg/kg of cannabis (7), which doses have been however demonstrated to increase the risk of road accidents. The manual motor speed was affected both with the dominant and the non-dominant hand in a group of SCs chronic users compared to controls (23). Attention and memory were impaired in SC users also, while attention functions were mostly preserved in cannabis use disorder (22, 24, 28), as evaluated by several tests such as the Digit Span Test, the Trial Making Test B, the Go/No Go Test, the Stroop test, and the Verbal Memory Processes Test. Executive functions (e.g., reaction time, commission and omission errors, impulse and reaction inhibition, changing strategies, mental flexibility, planning, visual spatial skills, organizing thoughts) and visual-spatial perception functions (e.g., Cube Drawing Test and Clock Drawing Test), usually conserved in cannabis users, were affected by SCs use disorder (22, 24, 28). This type of studies pointed toward an impairment of the mental flexibility, working memory and response inhibition in chronic SCs users (24, 28) and a general cognitive impairment as assessed by the Montreal Cognitive Assessment (MOCA) test (23). However, studies performed on SCs users did not test the concentrations in blood or urine and did not evaluate an acute effect (subjects were tested after 3 days of reported abstinence), but only the long-term one on cognitive functions, so that several confounding factors, e.g., treatment, past cannabis consumption history, other drugs consumption history, psychiatric diseases, abstinence time and withdrawal could have altered the results. On the basis of the studies involved in the present review, a risk for road accidents appear to involve both occasional and chronic SCs users. Regarding the clinical signs of impairment, the epidemiological data available in literature are in agreement with the experimental studies collected in this review. Among cases of DUI, a wider panel of SCs was detected on blood, including third generation SCs. Adamowicz and Lachowicz described a hyperactive behavior, gait abnormalities and staggering reported by the officers who first examined a SC consumer after a traffic accident. The accident occurred at 6:30 p.m., two and a half hours after the SC consumption (60). This data is consistent with the findings by Theunissen et al. describing the maximum psychomotor impairment within the first 2.5 h (21). It is interesting to note that the subjective high peaked 30 min after the administration, while a slowed response speed was seen until 4 h after consumption, suggesting a limited self-awareness of driving inability (21). Large variability in the subjective effects was also reported (26). In a Japanese paper collecting data from SC-related road accidents, “impaired consciousness” was described in most of the cases, with blurred vision, slowed motor activity, coordination and short memory impairment (64). Lemos et al. reported one case of DUI of the synthetic cannabinoid XLR-11 (quantified at 1.34 ng/ml). The driver was involved in a traffic collision, presented slurred speech, impaired attention and slowed movements, was unable to maintain balance and to perform the FTN correctly (56). Musshoff et al. described 7 cases of DUI with SCs, and the following findings were noted by the police or physicians visiting drivers: inability to follow basic instructions, vestibular disorders, delayed reactions of pupils to light, slowed movements, alterations in speech and in consciousness (61). Louis et al. reported 18 cases of DUI under the influence of SCs showing impairment of speech, coordination, eye convergence as well as alteration in the WAT and OLS (62). Another interesting finding was that SC-abused drivers might not remember the collision scene (64). Compared to cannabis user, persons arrested for driving under the influence of SCs were more confused, disorientated, incoherent, and showed slurred speech (83). Traffic offenses reported to SCs-intoxicated drivers ranged from erratic driving, lane traveling, speed driving, driving on curb, ignoring red lights until falling asleep, off roads, car accidents with or without pedestrian collisions (58, 63, 84). Similar findings, and psychomotor impairment, were noted from very low blood/serum/plasma levels, although the comparison between studies is limited by analytical issues as well as the different sampling times. Another point to consider is that NPS might be consumed in a setting of polydrug use, resulting in possible synergies and amplification of the effects (59, 61, 66, 84). In a recent paper, Funada et al. demonstrate that ethanol-induced motor impairments are enhanced when consumed with SC (85). However, further studies are needed to establish if and how much multiple consumption affects safe driving. Finally, studies performed on the road or by using an advanced driving simulator would bring a more truthful picture of the SC-related psychomotor impairment.

## Conclusions

Cases of DUI of SCs have been increasingly reported, although the exact prevalence of the phenomenon is likely to be underestimated. It is important to recognize signs of impairment through physical examination by trained and experienced personnel and to perform a thorough examination when there is a strong suspicion of SCs consumption, even when standard screenings test negative. Target LC-MS/MS methods are currently the most reliable technique for the detection and quantification of SCs in human specimens and should be preferred in these investigations. LC-QTOF-MS qualitative methods might also be helpful, allowing to search specifically for a wide range of new emerging substances. As demonstrated by animal and human experimental studies (from doses of 2-3 mg) and by studies on SCs users, SCs impair several psychomotor domains and psychomotor performances in humans, including motor performances, attention, memory, spatial memory, executive functions, and visual-spatial perception function. The impairment reported by these studies was confirmed by real cases of DUI of SCs, where subjects displayed a range from none to severe impairment (inability to stand, imbalance, lack of coordination, unconsciousness, etc.) with low SCs concentrations detected. Furthermore, several traffic offenses were reported and allowed to assess the effect of these substances on road, highlighting that SCs consumption represents a major problem for road safety.

## Data availability statement

The original contributions presented in the study are included in the article/Supplementary material, further inquiries can be directed to the corresponding author.

## Author contributions

AG, GB, and RG contributed to the conception, design of the study, and wrote sections of the manuscript. VO organized the database and wrote the first draft of the manuscript. AG supervised the final draft. All authors contributed to the manuscript revision, read, and approved the submitted version.

## Conflict of interest

The authors declare that the research was conducted in the absence of any commercial or financial relationships that could be construed as a potential conflict of interest.

## Publisher's note

All claims expressed in this article are solely those of the authors and do not necessarily represent those of their affiliated organizations, or those of the publisher, the editors and the reviewers. Any product that may be evaluated in this article, or claim that may be made by its manufacturer, is not guaranteed or endorsed by the publisher.
