# Supplementary material for: Effects of synthetic cannabinoids on psychomotor, sensory and cognitive functions relevant for safe driving
Source: Front Psychiatry. 2022 Sep 26;13:998828. doi: 10.3389/fpsyt.2022.998828 (PMC9548613; doi:10.3389/fpsyt.2022.998828)
Supplement: Supplementary file 1 [file Table_1.DOCX]

Supplementary Material

**Supplementary Table**

Tab.A Studies performed on animals

| FUNCTIONS | AUTHORS | YEAR | TESTING METHODS | TREATMENT | RESULTS |
| --- | --- | --- | --- | --- | --- |
| Locomotor activity | [52] | 2012 | Tetrad test battery | I.v. injections of 1-Pentyl-3-Phenylacetylindoles, (up to 30 mg/kg) | Suppression of locomotor activity |
| Locomotor activity; catalepsy | [50] | 2012 | Tetrad test battery | Inhalation of a mixture containing 5.4% JWH-018, trace amounts of JWH-073 and JWH-398 or Marijuana or placebo | Hypomotility, catalepsy, dose dependent ptosis and hyperreflexive responses after SC administration |
| Coordination; locomotor activity;  anxiety | [29] | 2013 | Elevated plus maze (EPM), hot-plate, open-field | I.p. injections of JWH-018, (0, 0.03, 0.1, or 0.3 mg/kg) | Reduction of locomotion reactivity, increased anxiety;  no major effects on motor coordination |
| Locomotor activity; catalepsy | [45] | 2014 | Tetrad test battery | I.p. injections of JWH-018 (0.01-6 mg/kg ) or Δ⁹-THC | JWH-018 induced spontaneous and handling-induced convulsions, hyperreflexia and myoclonias;  JWH-018 and Δ⁹-THC induced catalepsy; JWH-018 induced a prolonged and significant impairment in locomotion, Δ⁹-THC was effective only at the higher dose tested |
| Memory | [31] | 2014 | Novel object recognition task (NOR), spontaneous alternation on Y maze, spatial recognition memory on Y maze | I.p. injections of JWH-081 (0–1.25 mg/kg) or vehicle | JWH-081 impaired Object recognition, spontaneous alternation and spatial memory |
| Spatial memory; locomotor activity; exploratory activity;  anxiety levels | [32] | 2014 | Morris water maze, hole-board test | I.p. injections of CP55.940 (0.025, 0.125, 0.25 mg/kg) or vehicle | After a dose of 0.125 mg/kg: attenuated locomotor and exploratory activity, increased anxiety;  After a dose of 0.25 mg/kg: alteration in spatial memory |
| Locomotor activity | [43] | 2014 | Locomotor activity test | I.p. injection of Δ⁹-THC (1, 3, 10, 30 mg/kg), JWH-018 (0.03, 0.1, 0.3, 1 mg/kg), JWH-073 (0.3, 1, 3, 10 mg/kg), JWH-200 (0.3, 1, 3, 10 mg/kg), JWH-203 (0.3, 1, 3, 10 mg/kg), JWH-250 (1, 3, 10, 30 mg/kg), AM-2201 (0.1, 0.3, 1, 3 mg/kg), CP 47,497-C8-homolog (0.3, 1, 3, 10 mg/kg) or vehicle | Time and dose-dependent decrease of locomotor activity within the first 4 h |
| Locomotor activity; catalepsy | [35] | 2014 | Tetrad test battery | Inhalation (up to 100 mg/30L) and i.p. injections of JWH-018 (10 mg/kg), JWH-073 (30 mg/kg), Δ⁹-THC (100 mg/kg) | Catalepsy and dose-dependent impairment of locomotor activity, more potent following JWH-018 administration |
| Sensorimotor functions (visual, auditory, tactile); locomotion and neurological changes | [36] | 2015 | ANY-maze video tracking system, visual/acoustic/tactile sensorimotor tests | I.p. injections of JWH-018 (0.01-6 mg/kg) or Δ⁹-THC (1-6 mg/kg) | JWH-018 inhibited sensorimotion responses at lower doses, reduced spontaneous locomotion at intermiediate/high doses, induced convulsions, myoclonia and hyperreflexia at high doses |
| Locomotor activity; behavioural reactivity;  motor coordination; recognition memory | [54] | 2015 | Tetrad test battery, novel object recognition task (NOR) | I.p. injections of APICA (0, 1, 3 mg/kg) | Dose-dependent decrease of locomotor activity and behavioural reactivity; At the highest dose also motor incoordination, recognition memory impairment |
| General behaviour, locomotor activity, learning and memory | [51] | 2015 | Functional Observation Battery (FOB), locomotor activity test, rota-rod test, Morris water maze test | I.p. injections of JWH-081, JWH-210 (0.1, 1, 5 mg/kg) or vehicle (1 mg/kg) or (methamphetamine 5 mg/kg) | Traction and tremor after administration of 5 mg/kg of SCs; Locomotor activities significantly decreased; No significant changes in learning or memory functions |
| Locomotor activity; catalepsy | [53] | 2015 | Tetrad test battery | I.p. injections of Δ⁹-THC (5.6 mg/kg), AB-CHMINACA (3 mg/kg), AB-PINACA (30 mg/kg);  i.p. and i.v. injections of FUBIMINA (up to 100 mg/kg i.p.) | Dose-dependent suppression of spontaneous locomotor activity, with greater immobility time after AB-CHMINACA and AB-PINACA; FUBMINA suppressed locomotor activity and induced catalepsy only when administered i.v. |
| Locomotor activity; catalepsy;  visual, acoustic and tactile responses | [34] | 2016 | Tetrad test battery, visual object response, visual placing response, acoustic response test, vibrissae, observation of pinna and corneal reflexes | I.p. injections of JWH-250, JWH-073 (0.01–15 mg/kg) and co-administration of both drugs | Dose-dependent impairment of locomotor activity and visual/acoustic/tactile responses, up to 4-5 h at higher doses;  Co-administration impaired visual sensori-motor responses |
| Locomotor activity; catalepsy; aggressiveness; visual, acoustic and tactile responses | [30] | 2016 | Observation of tail elevation, hyperreflexia, myoclonus, convulsions, aggressive responses, visual placing response/visual object response, vibrissae/ pinna/corneal reflexes, tail pinch/tail withdrawal test, bar test, drag test, accelerod tests, spontaneous locomotor activity | I.p. injection of AKB48, 5F-AKB48, JWH-018 or Δ9 -THC (0.01–6 mg/kg) | SCs induced spontaneous and handling-induced convulsions, hyperreflexia and myoclonias, spontaneous and stimulated aggressiveness; Dose-dependent reduction of the visual object response; Transient reduction of the acoustic response;  Transient inhibition of the corneal reflex after administration of AKB48, deep inhibition after administration of 5F-AKB48; SC transiently inhibited the number of steps performed with the front legs, reduced the total distance travelled and increased the immobility time; AKB48 at 1 mg/ kg evoked a transient facilitation of spontaneous locomotion |
| Spatial memory; associative learning; general motor activity and anhedonia behaviour in adult subject repeatedly exposed to SC during adolescence | [37] | 2016 | Morris water maze, “NIR” video fear conditioning system, open field, tail suspension test | Daily i.p. injections of WIN55212.2 (0.5, 1, 3 mg/kg) during adolescence (3 weeks), tests during adolthood | Memory impairment in the Morris water maze;  Dose-dependent memory impairment in fear conditioning |
| Working memory; long-memory retention; locomotor activity; motivation in performing a motor task | [48] | 2016 | Novel Object Recognition (NOR), total distance travelled, average speed, total time of immobility, tail Suspension (TS) | I.p. injections of JWH-018 compounds (0.01-1 mg/kg) and Δ9 -THC (0.01-3 mg/kg) | SCs and Δ9-THC affected the distance travelled, the average speed and the immobility time;  SCs dose-dependently impaired both short and long-memory retention |
| Locomotor activity | [44] | 2016 | Locomotor activity test, drug discrimination test | I.p. injections of Δ9 -THC (2.5 – 25 mg/kg),ADBICA (0.25 – 2.5 mg/kg), ADB-PINACA (0.1 – 1 mg/kg), THJ-2201 (0.1 – 1 mg/kg), RCS-4 (1 – 10 mg/kg), JWH-122 (0.1 – 1.0 mg/kg), JWH-210 (0.5 – 5 mg/kg) or vehicle | All the compounds tested depressed locomotor activity, effects lasted 1.5 up to nearly 4 h |
| Spontaneous locomotor activity | [40] | 2017 | ANY-maze video tracking system | I.p. injections of JWH-018 (0.3-1 mg/kg) or AKB48 (0.3-1 mg/kg) or other drugs | JWH-018 induced long-lasting increases in the total distance traveled, the effects lasted 90 min; AKB48 facilitated the spontaneous locomotion only in the first 15 min after the injection; JWH-018 reduced the immobility time in mice, AKB48 increased it |
| Sensorimotor and neurological changes | [47] | 2017 | Visual/acoustic/tactile responses tests | I.p. injections of 5F‐ADBINACA, AB‐FUBINACA and STS‐135, (0.01–6 mg/kg) | Catalepsy;  Reduced motor activity;  Impaired sensorimotor responses (visual, acoustic and tactile);  Seizures, myoclonia, and hyperreflexia; promoted aggressiveness |
| Anxiety levels; spatial memory; exploratory and locomotor activity; neuromuscular performance and fatigue; depression | [49] | 2018 | Elevated plus maze, Y-maze and staircase paradigm, Gait balance and grip strength assessment, forced swimming test | I.p. injection of Δ⁹-THC, AB-FUBINACA, AB-CHMINACA and PB-22 (up to 25 mg/kg) | Decreased anxiety levels;  Spatial memory deficits;  Decreased exploratory behaviour; Decreased locomotor activity |
| Locomotor activity | [55] | 2019 | Locomotor activity test | Injections of Δ⁹-THC (2.5-25 mg/kg), AMB-FUBINACA (0.05-0.5 mg/kg), ADB-FUBINACA (0.05-1 mg/kg), 5F-MDMB-PINACA (0.133-1.33 mg/kg), MDMB-CHIMICA (0.01-0.05 mg/kg), MDMB-FUBINACA (0.01-0.1 mg/kg) or vehicle | Δ⁹-THC and AMB-FUBINACA: time and dose-dependent depression of locomotor activity within the first 10 min, maximal depression between 10-40 min and lasted up to 2.5 to 3 h at the highest dose; ADB-FUBINACA: time and dose-dependent depression of locomotor activity within the first 10 min, maximal depression between 0-30 min and lasted up to 2.5 h; 5F-MDMB-PINACA: time and dose-dependent depression of locomotor activity within the first 10 min, maximal depression between 0-30 min, return to baseline within 50 min; MDMB-CHIMICA: time and dose-dependent depression of locomotor activity, maximal depression between 10-40 min, return to baseline within 80-110 min; MDMB-FUBINACA: time and dose-dependent depression of locomotor activity within the first 10-20 min and lasted 30-60 min |
| Visual, acoustic and tactile responses; spontaneous locomotor activity | [46] | 2019 | Sensorimotor and locomotor tests, Pre-Pulse inhibition (PPI) test | I.p. injections of JWH-018-Cl, JWH-018-Br, AM-2201 (0.01-6 mg/kg) | Inhibition of sensorimotor and PPI responses at lower doses, reduced spontaneous locomotion at intermediate/high doses, inhibition of the pinnae reflex, reduction of the total distance travelled, increased immobility time; Dose-dependent reduction of the visual placing response, visual object response, acoustic response; AM-2201 and JWH-018-Cl significantly inhibited the corneal reflex |
| Anxiety, locomotor activity; recognition memory | [41] | 2019 | Open field (OF) test, novel object recognition test (NOR) | I.p. (0.003, 0.03, 0.3 mg/kg) and intracerebroventricular (10 nmol) injections of 5F-AMB | I.p. injection (0.3 mg/kg) dramatically decreased locomotor activity; intracerebroventricular infusion B (10 nmol) showed anxiolytic effect and impaired acquisition, but not retrieval, of recognition memory |
| Locomotor activity; catalepsy | [33] | 2019 | Tetrad test battery | Systemic administration of Δ⁹-THC (0, 10, 30 mg/kg), WIN55212-2 (0, 3, 10 mg/kg), XLR11 (0, 10, 30 mg/kg) | Catalepsy and dose-dependent locomotion impairment in wild type mice; Genetic deletion of CB1 receptors abolished the effects;  Genetic deletion of CB2 receptors abolished catalepsy by Δ⁹-THC and WIN55212-2, but not by XLR11; Generic deletion of GPR55 receptors caused enhanced responses to Δ⁹-THC or WIN55212-2 |
| Behavioural activity in adult subject repeatedly exposed to SC during adolescence | [39] | 2020 | Elevated plus maze (EPM), spontaneous alternation behaviour (SAB) in a Y-maze, novel object recognition test (NOR), marble burying (MB) | I.p. injections of 5F-MDMB-PICA (0.01, 0.01, 0.03 mg/kg) or vehicle | 5F-MDMB-PICA exposure during adolescence results in higher anxiety-like and compulsive-like state in adulthood |
| Locomotor activity; catalepsy | [38] | 2020 | Tetrad test battery | Injections of 5F-AKB48 (up to 3.2 mg/kg) or Δ⁹-THC | Cataleptic effects of 5F-AKB48 were more potent than those of Δ⁹-THC and lasted up to 3 h; More potent locomotor effects of 5F-AKB48 than Δ⁹-THC |
| Locomotor activity; catalepsy | [42] | 2020 | Tetrad test battery | I.p. injections of AB-FUBINACA (0-3 mg/kg) or vehicle | AB-FUBINACA (3 mg/kg) significantly increased catalepsy and immobility time |

i.v.: intravenous; i.p.: intraperitoneal. Δ⁹-THC: delta-9-tetrahydrocannabinol
